# The Dynamics of Condom Use with Regular and Casual Partners: Analysis of the 2006 National Sexual Behavior Survey of Thailand

**DOI:** 10.1371/journal.pone.0042009

**Published:** 2012-07-30

**Authors:** Aphichat Chamratrithirong, Paulina Kaiser

**Affiliations:** 1 Institute for Population and Social Research, Mahidol University, Phutthamonthon, Nakhon Pathom,Thailand; 2 Department of Epidemiology, School of Public Health, University of Michigan, Ann Arbor, Michigan, United States of America; Tulane University, United States of America

## Abstract

**Objective:**

This study aims to determine factors associated with levels of condom use among heterosexual Thai males in sex with regular partners and in sex with casual partners.

**Methods:**

The data used in this study are from the national probability sample of the 2006 National Sexual Behavior Study, the third nationally representative cross-sectional survey in Thailand. A subtotal of 2,281 men were analyzed in the study, including young (18–24) and older (25–59) adults who were residents of rural areas of Thailand, non-Bangkok urban areas, and Bangkok. Two outcomes of interest for this analysis are reported condom use in the past 12 months by males in relationships with the most recent regular and casual partners who were not sex workers. Chi-square statistics, bivariate regressions and the proportional odds regression models are used in the analysis.

**Results:**

Condom use for men with their regular partner is revealed to be positively related to education, knowledge of condom effectiveness, and pro-condom strategy, and negatively related to non-professional employment, status of registered marriage, and short relationship duration. Condom use with casual partner is positively determined by education, condom knowledge, non-professional occupation, short relationship duration, and lack of history of paid sex.

**Conclusion:**

The national survey emphasized the importance of risk perceptions and condom motivations variables in explaining condom use among men in Thailand. These factors include not only education and knowledge of condom effectiveness and pro-condom strategy but also types of partners and their relationship context and characteristics. Program intervention to promote condom use in Thailand in this new era of predominant casual sex rather than sex with sex workers has to take into account more dynamic partner-based strategies than in the past history of the epidemics in Thailand.

## Introduction

Promoting condom use has been a key intervention in preventing the spread of HIV and other sexually transmitted infections (STIs). When used consistently, condoms can be up to 95% effective in preventing HIV transmission; consistent condom users are 10 to 20 times less likely to be infected after exposure to HIV than inconsistent or non-users [1]. However, condom use remains highly varied around the world. One of the strongest determinants of condom use is relationship type: condom use is typically high with commercial sex partners but exceedingly low with spouses or regular partners [2]. Thailand provides a case study that exemplifies such a pattern. Improving knowledge of the factors associated with condom use, particularly among regular and casual partners – in sex with regular partners and in sex with non-regular partners – is a critical step towards increasing condom use and decreasing transmission of HIV and other STIs.

### Factors that Affect Condom Use

#### Demographic and relationship factors

Demographic factors such as age, gender, education, and urbanicity have been linked to condom use in multiple previous studies. In many countries, men are more likely to report condom use than women, in part because men are more likely to have sex with casual partners and/or sex workers with whom condom use is more common [3]. Age is also important in understanding patterns of condom use; younger people are more likely to have better knowledge of condoms and may be more likely to use condoms [4]. Urbanicity and higher education have also been associated with better knowledge of condoms [4].

Condom use in committed partnerships is often very rare; for example, marital status was the strongest predictor of condom use among women in Uganda, with currently married women least likely to report condom use at last sex [4]. Relationship characteristics, such as the duration of a relationship and the frequency of new relationships, also affect condom use; Ku and colleagues illustrated the ‘sawtooth hypothesis’ of condom use, where condom use declines as relationships lengthen and successive relationships are less likely to begin with condom usage [5]. Further, previous research in Madagascar has explored the fluidity between paid sex interactions and personal relationships, with subsequent effects on condom use [6].

#### Condom factors

Other factors, such as social norms around condoms and condoms’ impact on male pleasure, are commonly provided reasons for lack of condom use. Multiple studies from Thailand have documented perceptions among men that condom use reduces pleasure of sexual intercourse [7]. Therefore, use of condoms requires a compelling reason – such as fear of HIV infection – to override the loss of pleasure [8].

The availability of other methods of contraception, with fewer perceived drawbacks than condoms, may also explain unwillingness to use condoms. In 2000, the most common contraceptive method used in Thailand was the pill (26.8% of women), followed by female sterilization (22.6%); condom use was uncommon as a main contraceptive method (1.7%) [9].

Another common explanation for the lack of condom use in regular partnerships is the perception that condoms are primarily associated with disease prevention rather than contraception. In regular partnerships, condom use is typically higher when one partner is known to be high-risk than when neither partner acknowledges high-risk status [10]. Also, condom promotion interventions among sex workers and their clients tend to be more successful than condom promotion interventions for committed relationships [11]. More than one-third of respondents to a 1990 survey in Thailand agreed that asking to use a condom with a regular partner is insulting to the partner, due to the insinuation that condoms are only necessary when risk of disease transmission exists [8].

Access to condoms is a key prerequisite for condom use, but one that remains understudied. The 2006 National Sexual Behavior Survey of Thailand (the data used for this analysis) found that the second most common reason provided by men who did not use a condom at last sex with a casual partner was ‘not prepared/could not find a condom at the time’ [12]. It was found that increased access to condoms was associated with higher intentions to use condoms in a study of South African secondary students [13]. Condom access has been improved in many areas through the use of social marketing campaigns, which serve to increase availability and decrease stigma [14], [15].

### HIV and Condoms in Thailand

Thailand provides a unique setting for the study of HIV and condoms due to the fact that the first HIV case in Thailand was identified in 1985, and the first indigenous transmission was documented in 1987 [16]. The Thai HIV epidemic was first identified in injecting drug users, but quickly spread to commercial sex workers [17]. Sex workers in Thailand are largely brothel-based; though sex work is illegal, it has a stable existence in Thai society [8]. In 1989, 3.1% of brothel-based sex workers were HIV-positive; by 1994, the proportion had risen to 31% [14]. In 1991, the Thai government implemented a national program to encourage condom use in all sexual encounters with commercial sex workers [17]. The 100% Condom Use program included provision of free condoms to commercial sex establishments, sanctions against establishments that did not use condoms consistently, and a media campaign to provide HIV education and encourage condom use with sex workers [18]. Additionally, multiple large research studies in Thailand have explored knowledge, attitudes, and practices in relation to HIV and sexual behavior [8].

As a result of the condom promotion program, condom use with sex workers in Thailand jumped from 14% in 1989 to 95% in 1993 [19]. From 1989 to 2000, the number of STIs in Thailand plummeted by more than 95% [20]. By 1996, the program may have prevented more than 2 million HIV infections [21]. The 100% Condom Use program has been lauded around the world as a model of a cost-effective intervention to prevent HIV and STIs [20], [11].

While condom use with sex workers is common in Thailand, condom use is inconsistent with casual partners and extremely rare among married couples [22]. Only 21% of sexually active Thai high school students reported ever having used condoms [23]. For most recent intercourse, 27% of high school men but just 0.5% of high school women reported using a condom [24]. Qualitative research has found that the main barriers to condom use are interference with male sexual pleasure and the perception of condoms as prophylaxis for use with prostitutes [8].

Thailand’s unique cultural and historical context contributes to a setting with varied levels of condom use despite the presence of HIV and substantial government intervention. As HIV transmission due to commercial sex declines, thanks to the success of the 100% Condom Use program, the relative importance of HIV transmission through casual and regular partners increases. Therefore, the research aim of this analysis is to determine which factors are associated with higher levels of condom use among heterosexual Thai males in sex with regular partners and in sex with casual partners.

## Methods

The 2006 National Sexual Behavior Study (NSBS) provides the data used in this analysis. The data were collected by the Institute for Population and Social Research at Mahidol University in Bangkok, Thailand with support from UNAIDS and the UN Thailand Country Team. The 2006 NSBS is the third nationally representative cross-sectional study in Thailand to track sexual behaviors as well as knowledge and attitudes related to HIV/AIDS. The respondents were between 18 and 59 years of age. The consent form was read and explained to them by the interviewers. If the respondents agreed to participate in the survey, the interviewers would sign their name in the informed consent form for the record, indicating that they had informed the respondents and the respondents had verbally given informed consent to take part in the study. The respondents would not sign their name in the informed consent form because in Thailand the respondents would not be comfortable to sign document. The study protocol including all these data collection and consent procedures were reviewed and approved (on condition that the study would not involve respondents under 18 years of age), by the Institutional Review Board of the Institute for Population and Social Research, Mahidol University, Bangkok (in which the authors of this study were not a member of the IRB committee.).

### Data Collection

The national probability sample ensured equal participation from men and women, young adults (18–24) and older adults (25–59), and residents of rural areas, non-Bangkok urban areas, and Bangkok. To recruit non-Bangkok urban and rural participants, 14 provinces (out of 75 in the country) were selected randomly, with selection probability proportional to population size. Within each selected province, two districts were selected; within each district, rural and urban areas were enumerated. Fourteen of the enumerated urban areas were selected, from which four election districts were randomly selected, with nine interviews per age/sex stratum completed within each election district. Among the enumerated rural areas, sub-districts were identified and three villages in each sub-district were selected (proportional to population size). For each village, a complete household listing was obtained, and three interviews were completed for each age/sex strata. In Bangkok, 63 election districts were randomly selected, and four households were systematically selected from each district.

The survey was completed in-person with sex-matched interviewers. Interviewer teams composed of two male and two female interviewers, with one interviewer assigned to each age/sex stratum (young male, young female, older male, older female), were sent to each geographical location for data collection. In all geographical areas, the interviewers were sent to different households; all household members were listed by sex and age group. The interviewer then recruited a household member in the age/sex strata assigned to that interviewer. If such a person lived in the household but was not immediately available, the interviewers made appointments to come back; if there was no appropriate person in the household, the interviewer moved to the household to the left.

A total of 6,048 surveys were completed, of which exactly half (3,024) were completed by men; each strata of age (18–24 and 25–59), gender (male and female), and location (Bangkok, non-Bangkok urban, and rural) contained 504 responses. The overall response rate was 81%, with higher rates among young adults (89%) than older adults (73%).

### Variables of Interest

#### Condom use

There are two outcomes of interest for this analysis: reported condom use by males in relationships with regular partners and reported condom use by males in relationships with casual partners. The survey instrument assessed frequency of condom use separately for the most recent regular partner and the most recent casual partner in the past 12 months. The response options provided were ‘never,’ ‘sometimes,’ ‘about half the time,’ ‘mostly,’ and ‘always.’ For analysis, reported condom use was condensed into a three-level variable with categories of never, sometimes/about half, and mostly/always. Using a three-level outcome preserved statistical efficiency, especially with the smaller sample size of men with casual partners, while preserving the distinctions between different amounts of condom use.

However, logistic regression analysis was conducted (results not shown in this study) using dichotomous outcomes of condom use, designed according to the difference of condom use distribution among the two groups (regular and casual partners). We used dichotomous outcome of any condom use vs. never use condom for regular partners, and we used condom use with half or more sex acts vs. use condom sometimes or never use condoms for casual partners. The differences between these analyses and the analysis with three level categories mentioned above, were found to be small and generally do not change the interpretation of the results. We decided that in order to simplify the analysis, the logistic regression analysis mentioned here will not be presented other than to describe the few differences.

#### Partner type

Regular partners were defined as a partner with whom the respondent had sex for a period of one year or more, or, if the relationship was less than one year, a sexual relationship expected to continue in the future. Casual partners were not regular partners who were not sex workers.

#### Demographic and socioeconomic status

The demographic and socioeconomic status variables includes age, geographical location, education, occupation type, and marital status. For analysis, age was included as a continuous variable. Geographical location (used in the sampling frame of the NSBS) was collected as Bangkok, other urban, and rural. Respondents from Bangkok and from other urban areas demonstrated similar patterns in condom use as well as other variables of interest, so geographic location was reduced to urban and rural categories only. Education level was recorded as the highest level of schooling completed. Education levels were condensed in order to maximize power. Occupation type was categorized to professional, sales/service, skilled technical, labor, and unemployed. Among men with casual partners, occupation categories were reduced to high-skill (professional and skilled technical) and low-skill (labor and sales/service) due to their similarities in association with condom use and in order to maximize statistical efficiency. Marital status was collected as unmarried, married and registered, married but not registered, and widowed/divorced/separated. Due to low numbers of widowed, divorced, or separated men, marital status was re-coded to three groups: single, married and registered, and married and not registered. Marital status was not included in the analysis of men with casual partners since 84% of these men were unmarried. When marriage is registered the partnership is believed to be more serious than just living together where men usually will still consider themselves as single. Marriage without registration is mostly custom marriage which is considered to be also more serious than single persons living together as regular partners. The attachment of marriage with this degree of seriousness is hypothesized to be more related to trust and fidelity.

#### Access to condoms

The survey included the question, “In your community or workplace, is there a place to distribute free or low-price condoms?” For analysis, responses were dichotomized to ‘yes’ vs. all other responses (‘no,’ ‘not sure,’ and ‘don’t know’).

#### Condom knowledge and attitudes

Condom knowledge was measured by asking an open-ended question about which actions could prevent someone from contracting HIV. Respondents who mentioned using a condom without prompting from the interviewer were considered to have knowledge of the HIV-preventive benefits of condoms. Attitude towards condoms was measured by asking participants to select the AIDS-prevention strategy they would choose, among reducing sexual activity, using condoms consistently, or both. Respondents who chose using condoms consistently or using both strategies were considered to have pro-condom attitudes.

#### Partner characteristics

The total number of partners (regular, casual, partners with whom things or favors are exchanged for sex, and partners with whom money is exchanged for sex) reported in the past 12 months was calculated and dichotomized into categories of ‘one partner’ and ‘more than one partner.’ The duration of the most recent relationship in the past 12 months was categorized as 30 days or less, 31 to 90 days, and more than 90 days. Men were asked if they had ever given money in exchange for sex; responses were categorized as never, more than a year ago, and within the past year.

### Analysis

This analysis included only males, because a very high proportion of Thai females report just one lifetime sexual partner (their spouse) with whom condoms are rarely used, while males report more variation in type of partners and in condom use [12]. Of the 3,024 men participating in the survey, 377 were excluded for reporting no history of sexual activity, 28 for reporting sexual attraction to males, and 313 for not having a casual or regular partner in the past 12 months. An additional 24 cases were excluded due to missing information relating to condom access, knowledge, or attitudes. The analytic dataset includes 2,281 men, of whom 1,998 contribute to the analysis of regular partners and 520 contribute to the analysis of casual partners. Two hundred thirty-seven men contribute to both analysis sets. All analyses were completed using SAS version 9.2 [25].

Since the sampling design intended to capture a nationally representative sample, the data were weighted to national demographic characteristics. Chi-square statistics were used to evaluate the differences between proportions among levels of each covariate. Bivariate regressions provided the crude association between condom use (with regular partners and with casual partners) and each of the predictor variables. The proportional odds models were built by examining socio-demographic factors and condom/partner factors separately and then together; variables were eliminated from the full model based on statistical significance and tests of the difference of -2 times the log likelihood. The score test for the proportional odds assumption was used to check the fit of the proportional odds models; the validity of the proportional odds assumption was also verified by manual calculation of odds ratios using different dichotomous cutpoints in the categorization of condom use (analysis not shown here). Possible interactions between socio-demographic factors and condom/partner factors were examined, and collinearity between variables was evaluated; no notable results were found.

For casual partners, models were constructed predicting both high condom use and low condom use. Since most men with casual partners reported some level of condom use, predicting low condom use provided slightly more power and smaller confidence intervals, but did not change the magnitude of effects or the variables included in the final model. Therefore, for clarity of presentation, we present results for predicting high condom use among men with casual partners and with regular partners.

As for the conceptual framework for the analysis, the independent variables considered will be classified into two groups as well as demographic characteristics as control variables. The two groups are 1) “Risk perception” - factors related to perceptions of the risks of HIV/AIDS and STD, and 2) “Condom motivations” - factors related to the motivation to take preventive action by using condom. Risk perception variables include education, marital status, number of partners in the past 12 months, duration of relationship or the newness of partners, and the experience of giving money for sex. Condom motivations variables include unprompted knowledge of condom effectiveness in HIV prevention, attitude of condom use as chosen strategy to reduce HIV risk, and self report access to convenient and cheap condoms. As for the control variables which are also used to address the possible bias due to the selectivity of men engaging in regular or casual relations, these variables are age, location (urban/rural) and occupation.

## Results

The study population consisted of men with an average age of 32 (median 28; mode 18), ranging from 18–59. Overall, 15% of the sample had less than a fourth grade education, while 13% were educated beyond high school. Almost all men with less than a fourth grade education were over 35. More than one-fifth of men (22.2%) reported having more than one sexual partner in the past year. Employment in a skilled technical field was most common (30.1% of men), while 17.6% were unemployed. More than half of the sample (59.5%) were married (35.1% registered and 24.4% not registered).

Descriptive analysis (see Table 1) revealed differences between men with regular partners and men with casual partners. The weighted mean age among men reporting having regular partners was 33.7 (SD: 11.2); men with casual partners were younger, with a mean age of 27.4 (SD: 6.8). Almost half of men with casual partners were aged 18–24 (46.9%).

**Table 1:** Characteristics of study population: men with at least one regular or casual sex partner in the past 12 months in 2006 the National Sexual Behavior Survey of Thailand (N = 2,281).

	*Men with regular partners (N = 1998)*			*Men with casual partners (N = 520)*		
	*Unweighted N*	*Weighted Frequency*	*p-value*	*Unweighted N*	*Weighted Frequency*	*p-value*
**Sociodemographic**						
Location						
*Bangkok*	710	10.7%		197	14.2%	
*Other urban area*	656	29.5%		180	36.7%	
*Rural area*	632	59.9%	<0.0001	143	49.1%	<0.0001
Occupation						
*Unemployed*	304	7.4%		168	20.1%	
*Professional*	267	12.5%		70	17.2%	
*Sales/service*	446	19.9%		92	20.7%	
*Skilled technical*	648	44.1%		111	30.1%	
*Labor*	333	16.2%	<0.0001	79	11.9%	<0.0001
**Risk perception factors**						
Education						
*Less than grade 4*	339	23.9%		12	3.6%	
*Grade 5–7*	407	23.9%		79	17.0%	
*Junior high school*	486	18.4%		200	34.6%	
*Senior high school*	494	20.1%		178	33.6%	
*Vocational/BA or higher*	272	13.8%	<0.0001	51	11.3%	<0.0001
Marital status						
*Single*	620	13.8%		427	70.0%	
*Married & registered*	800	54.2%		29	8.6%	
*Married, not registered*	556	30.6%		52	15.7%	
*Widowed/divorced/separated*	22	1.4%	<0.0001	12	5.7%	<0.0001
Number of partners in past 12 months						
*One*	1647	89.0%		128	23.3%	
*More than one*	351	11.0%	<0.0001	392	76.7%	<0.0001
Ever gave money for sex						
*Yes – within the past year*	136	6.8%		108	20.8%	
*Yes – more than a year ago*	600	30.0%		113	21.7%	
*No*	1262	63.2%	<0.0001	299	57.5%	<0.0001
Duration of relationship						
*30 days or less*	401	20.1%		225	43.3%	
*31–90 days*	275	13.8%		151	29.0%	
*More than 90 days*	1322	66.2%	<0.0001	144	27.7%	<0.0001
**Condom motivation factors**						
Self-reported access to convenient & cheap condoms	561	33.5%	–	160	35.7%	–
Unprompted knowledge of condom effectiveness in HIV prevention	1491	67.2%	–	458	83.9%	–
Condom use as chosen strategy to reduce HIV risk	783	31.7%	–	369	69.0%	–
**Frequency of condom use**						
Always	146	4.2%		307	60.1%	
Mostly	93	2.4%		33	5.3%	
About half	41	1.3%		8	1.1%	
Sometimes	450	18.0%		80	13.1%	
Never	1268	74.1%	<0.0001	92	19.8%	<0.0001

Men with casual partners had more partners in the past 12 months than men with regular partners (casual, 3.6 [SD 3.7]; regular, 1.6 [SD 2.09]). Among men with regular partners, the majority (91.8%) had just one regular partner and no casual partners in the past 12 months. Few men (5.8%) had one regular partner and one or more casual partners, while 2.4% had more than one regular partner. Among men with casual partners, slightly more than half (55.2%) had no regular partners, and 53.1% had just one casual partner in the past 12 months. More than one-third (39.2%) had one regular partner and one or more casual partners in the past 12 months.

### Men with Regular Partners

In bivariate analysis, all socioeconomic factors and condom-related factors that we considered were associated with condom use (p<0.05; see Table 2). Increased age was associated with decreased use of condoms, while urban residence was associated with increased odds of reporting higher levels of condom use. Increasing education displayed a strong trend (Cochran-Armitage Trend Test, p<0.0001) with increasing levels of education associated with increased use of condoms, such that compared to men with less than four years of education, men with post-high school education were 13 times more likely to reported higher levels of condom use. The professionals are found to use condoms more than any other occupation except for the unemployed. Especially those who were employed as labor, skilled and technical, and sales/service workers were less likely than the professionals to report the higher levels of condom use. The attachment of marriage with the higher degree of seriousness is found to be related to condom use. Being married and registered was associated with a ten-fold reduction in the odds or reporting higher levels of condom use; being married but not registered was associated with a six-fold decrease in the odds of reporting higher levels of condom use. Men in regular partnerships who reported having access to condoms were slightly less likely to report using condoms than men without access to condoms. Condom knowledge and pro-condom strategy choice were both associated with more than double the odds of higher levels of condom use, as was having more than one partner in the past twelve months. Duration of relationship did not have a significant effect on condom use. While recent (within the past year) payment for sex was associated with increased likelihood of reporting higher levels of condom use, payment for sex in the long past (more than a year) decreased the condom use.

**Table 2:** Unadjusted and adjusted odds ratios of reporting greater condom use (never, sometimes/half, mostly/always) among men with regular partners (N = 1,998).

	*Unadjusted*		*Adjusted*	
	*Odds Ratio*	*95% CI*	*Odds Ratio*	*95% CI*
**Sociodemographic factors**				
Age (continuous)	0.91***	0.90–0.92	0.95***	0.94–0.97
				
Location				
*Rural*	1	(ref)	–	
*Urban*	1.78***	1.47–2.16	–	
Occupation				
*Professional*	1	(ref)	1	(ref)
*Skilled technical*	0.33***	0.25–0.44	0.63*	0.45–0.87
*Sales/service*	0.58***	0.42–0.79	0.74	0.53–1.04
*Labor*	0.29***	0.20–0.42	0.51*	0.34–0.77
*Unemployed*	1.68**	1.16–2.44	1.00	0.65–1.53
**Risk perception factors**				
Education				
*Less than grade 4*	1	(ref)	1	(ref)
*Grade 5–7*	2.88***	1.91–4.34	1.32	0.84–2.07
*Junior high school*	6.73***	4.52–10.03	2.23***	1.44–3.57
*Senior high school*	8.40***	5.68–12.41	2.61***	1.67–4.09
*Vocational/BA or higher*	13.04***	8.70–19.55	4.54***	2.86–7.20
Marital status				
*Unmarried*	1	(ref)	1	(ref)
*Married & registered*	0.10***	0.08–0.13	0.30***	0.22–0.42
*Married, not registered*	0.16***	0.12–0.21	0.33***	0.24–0.45
				
More than one partner in past 12 months	2.99***	2.29–3.89	–	
Ever gave money for sex				
*No*	1	(ref)	–	
*Yes – more than a year ago*	0.70**	0.57–0.87		
*Yes – within the past year*	2.64***	1.78–3.93		
Duration of relationship				
*More than 90 days*	1	(ref)	1	(ref)
*31–90 days*	1.21	0.92–1.59	0.83	0.61–1.13
*30 days or less*	0.87	0.68–1.31	0.68**	0.51–0.91
**Condom motivation factors**				
Self-reported access to convenient & cheap condoms	0.81*	0.66–1.00	–	
Unprompted knowledge of condom effectiveness in HIV prevention	2.35***	1.87–2.95	1.41*	1.09–1.81
Condom use as chosen strategy to reduce HIV risk	2.25***	1.85–2.74	1.38*	1.10–1.72

* p<0.05

** p<0.01

*** p<0.001.

Adjusting for socio-demographic factors in the multinomial proportional odds model moderated the effect of condom and partner factors (see Table 3). The final multinomial model retained age, education (with a trend still clear and as expected, but not statistically significant), occupation, marital status, condom knowledge, pro-condom strategy, and relationship duration. It should be noted that recent payment for sex (in the past year) remained to increase the likelihood of condom use when logistic regression analysis was conducted (Tables not shown here) using dichotomous outcome of any condom use vs. never use condoms. The largest magnitude of effect is observed for education level, particularly with high levels of completed education. Being married retained its substantial association with reduced levels of condom use. Shorter relationships (30 days or less, compared to more than 90 days) were associated with lower levels of reported condom use. The final model explained approximately one-third of the variance in condom use (R^2^ = 0.31).

**Table 3:** Unadjusted and adjusted odds ratios of reporting greater condom use (never, sometimes/half, mostly/always) among men with casual partners (N = 520).

	*Unadjusted*		*Adjusted*	
	*Odds Ratio*	*95% CI*	*Odds Ratio*	*95% CI*
**Sociodemographic factors**				
Age (continuous)	1.02	0.99–1.05	1.04*	1.00–1.07
Location				
*Rural*	1	(ref)	-	
*Urban*	1.21	0.77–1.90	-	
Occupation				
*Professional/technical*	1	(ref)	1	(ref)
*Sales/service/labor*	2.40**	1.38–4.17	2.57**	1.41–4.68
*Unemployed*	1.14	0.64–2.03	0.99	0.51–1.94
**Risk perception factors**				
Education				
*Less than grade 7*	1	(ref)	1	(ref)
*Junior high school*	1.38	0.63–3.08	1.52	0.80–2.91
*Senior high school*	3.14***	1.64–6.02	3.58***	1.78–7.22
*Vocational/BA or higher*	1.39	0.63–3.08	1.43	0.61–3.32
More than one partner in past 12 months	1.55	0.93–2.59	-	
Ever gave money for sex				
*No*	1	(ref)	1	(ref)
*Yes – more than a year ago*	1.05	0.66–1.69	0.44**	0.24–0.79
*Yes – within the past year*	0.98	0.60–1.62	0.78	0.41–1.47
Duration of relationship				
*More than 90 days*	1	(ref)	1	(ref)
*31–90 days*	3.11	1.85–2.51	1.97*	1.09–3.56
*30 days or less*	2.54^****^	1.54–4.19	2.22**	1.24–3.98
**Condom motivation factors**				
Self-reported access to convenient & cheap condoms	1.09	0.68–1.75	-	
Unprompted knowledge of condom effectiveness in HIV prevention	1.84*	1.03–3.29	2.36**	1.26–4.43
Condom use as chosen strategy to reduce HIV risk	1.04	0.64–1.68	-	

* p<0.05

** p<0.01

*** p<0.001.

### Men with Casual Partners

Due to the smaller sample size, many fewer factors were associated with condom use among men with casual partners (see Table 4). Age had a very small positive effect on condom use, while urban residence did not affect condom use. Moderate levels of education increased the odds of reporting higher levels of condom use, reaching significance for men with a senior high school education compared to those with less than a seventh-grade education. The relationships are also found among the junior high and BA to be positive as expected, but not statistically significant. This may be because of the small number of cases among these two groups. It is also possible that apart from formal education, “skill” and “informal training” may also be important in condom use behavior. Contrary to the finding among regular partnerships, men employed in labor, sales, or service jobs are more likely to use condoms with causal partners than the professional/technical occupation counterparts.

**Table 4:** Building the final proportional odds regression model to predict higher levels of condom use for men with regular partners in the 2006 National Sexual Behavior Survey of Thailand (N = 1,998).

	*Sociodemographic model*		*Condom/partner model*		*Full model*		*Final model*	
	*Odds Ratio*	*95% CI*	*Odds Ratio*	*95% CI*	*Odds Ratio*	*95% CI*	*Odds Ratio*	*95% CI*
**Sociodemographic**								
Age	0.95***	0.94–0.96			0.95***	0.94–0.97	**0.95** ***	**0.94–0.97**
Rural residence	1	(ref)			-		**-**	
Urban residence	1.19	0.96–1.49						
Less than grade 4	1	(ref)			1	(ref)	**1**	**(ref)**
Grade 5–7	1.33	0.95–2.08			1.29	0.82–2.02	**1.31**	**0.84–2.06**
Junior high school	2.19***	1.39–3.45			2.18***	1.38–3.44	**2.21** ***	**1.40–3.49**
Senior high school	2.59***	1.66–4.05			2.50***	1.59–3.92	**2.58** ***	**1.65–4.04**
Vocational/BA or higher	4.48***	2.82–7.10			4.38***	2.75–6.96	**4.45** ***	**2.81–7.07**
Professional	1	(ref)			1	(ref)	**1**	**(ref)**
Skilled technical	0.64**	0.46–0.89			0.62*	0.45–0.86	**0.62** *	**0.42–0.92**
Sales/service	0.72	0.51–1.01			0.74	0.52–1.04	**0.74**	**0.53–1.04**
Labor	0.51**	0.34–0.77			0.50**	0.33–0.76	**0.63** **	**0.45–0.87**
Unemployed	1.03	0.67–1.58			1.03	0.67–1.58	**1.00**	**0.62–1.53**
Unmarried	1	(ref)			1	(ref)	**1**	**(ref)**
Married & registered	0.29***	0.21–0.39			0.32***	0.23–0.45	**0.30** ***	**0.22–0.42**
Married, not registered	0.32***	0.23–0.43			0.34***	0.25–0.46	**0.33** ***	**0.24–0.45**
**Condom/partner**								
Condom access			0.83	0.67–1.02	-		**-**	
Condom knowledge			2.06***	1.64–2.60	1.38*	1.07–1.78	**1.41** **	**1.09–1.81**
Pro-condom strategy			1.82***	1.49–2.24	1.34*	1.07–1.68	**1.38** **	**1.10–1.72**
More than one partner			2.25***	1.58–3.20	1.04	0.71–1.53	**-**	
Relationship duration more than 90 days			1	(ref)	1	(ref)	**1**	**(ref)**
Relationship duration 31–90 days			0.90	0.67–1.21	0.79	0.57–1.09	**0.83**	**0.61–1.13**
Relationship duration 30 days or less			0.69**	0.53–0.91	0.65**	0.48–0.87	**0.68** **	**0.51–0.91**
Never gave money for sex			1	(ref)	1	(ref)	**-**	
Gave money for sex more than a year ago			0.69***	0.56–0.86	1.04	0.81–1.32		
Gave money for sex within the past year			1.03	0.62–1.72	1.71	0.99–2.95		
R^2^	0.2981		0.0981		0.3105		**0.3077**	
-2 log L	2620.948		2998.819		2595.001		**2598.505**	

* p<0.05

** p<0.01

*** p<0.001.

Unprompted knowledge of condom effectiveness in preventing HIV transmission was associated with higher levels of condom use. Shorter relationships were also associated with higher levels of condom use than relationships lasting more than 90 days. Finally, paying for sex more than a year ago was associated with lower likelihood of using condoms in a current relationship. Condom access and having more than one partner in the past year had moderately positive effects on condom use, but did not reach significance.

The most parsimonious proportional odds model contained age, education, occupation, condom knowledge, relationship duration, and history of paid sex (Table 5). However, these variables explained less of the variation in condom use (R^2^ = 0.14), compared to the model on regular partners.

**Table 5:** Building the final proportional odds regression model to predict higher levels of condom use for men with casual partners in the 2006 National Sexual Behavior Survey of Thailand (N = 520).

	*Sociodemographic model*		*Condom/partner model*		*Full model*		*Model 4*		*Final model*	
	*Odds Ratio*	*95% CI*	*Odds Ratio*	*95% CI*	*Odds Ratio*	*95% CI*	*Odds Ratio*	*95% CI*	*Odds Ratio*	*95% CI*
**Sociodemographic**										
Age	1.03	0.99–1.06			1.04*	1.00–1.07	1.04*	1.00–1.07	**1.04** *	**1.00–1.07**
Rural residence	1	(ref)			1	(ref)	-		**-**	
Urban residence	0.95	0.58–1.54			0.95	0.56–1.60				
Less than grade 7	1	(ref)			1	(ref)	1	(ref)	**1**	**(ref)**
Junior high school	1.56	0.83–2.94			1.43	0.73–2.81	1.39	0.72–2.68	**1.52**	**0.80–2.91**
Senior high school	3.56***	1.76–7.16			3.68***	1.76–7.70	3.50***	1.72–7.12	**3.58** ***	**1.78–7.22**
Vocational/BA or higher	1.62	0.70–3.75			1.48	0.61–3.60	1.43	0.60–3.40	**1.43**	**0.61–3.32**
Professional/technical	1	(ref)			1	(ref)	1	(ref)	**1**	**(ref)**
Labor/sales & service	2.37**	1.34–4.20			2.55*	1.39–4.70	2.54*	1.39–4.65	**2.57** *	**1.41–4.68**
Unemployed	1.04	0.55–1.97			0.97	0.50–1.91	0.97	0.50–1.90	**0.99**	**051–1.94**
**Condom/partner**										
Condom access			1.41	0.85–2.33	1.49	0.87–2.57	1.52	0.89–2.57	**-**	
Condom knowledge			1.89*	1.03–3.64	2.41**	1.28–4.57	2.43**	1.29–4.58	**2.36** **	**1.26–4.43**
Pro-condom strategy			1.00	0.61–1.66	0.89	0.52–1.53	-		**-**	
More than one partner			1.59	0.90–2.83	1.51	0.83–2.74	1.52	0.84–2.75	**-**	
Relationship duration more than 90 days			1	(ref)	1	(ref)	1	(ref)	**1**	**(ref)**
Relationship duration 31–90 days			2.07***	1.17–3.69	2.06*	1.11–3.80	2.08*	1.13–3.82	**1.97** *	**1.09–3.56**
Relationship duration 30 days or less			2.63*	1.49–4.66	2.34**	1.29–4.25	2.30**	1.27–4.17	**2.22** **	**1.24–3.98**
Never gave money for sex			1	(ref)	1	(ref)	1	(ref)	**1**	**(ref)**
Gave money for sex more than a year ago			0.53*	0.30–0.92	0.41**	0.22–0.74	0.41**	0.22–0.74	**0.44** **	**0.24–0.79**
Gave money for sex within the past year			0.73	0.38–1.39	0.63	0.32–1.25	0.65	0.33–1.27	**0.78**	**0.41–1.47**
R^2^	0.0773		0.0672		0.1492		0.1486		**0.1383**	
-2 log L	539.133		542.772		512.287		512.511		**516.445**	

* p<0.05

** p<0.01

*** p<0.001.

## Discussion

This analysis, utilizing data from a national survey of sexual behavior in Thailand, emphasizes the importance of education in determining condom use in regular partnerships and in casual partnerships; among men with regular partners and men with casual partners, higher levels of education are associated with higher levels of condom use. However, condom-specific knowledge is also found to have an impact distinct from years of schooling, particularly for men in casual partnerships. Self-reported condom access was not associated with condom use among men with regular partners, but may have a moderate effect on condom use among men with casual partners (though the effect did not reach significance in this analysis, which was constrained by limited sample size). Employment type and duration of relationship were important in explaining condom use in both men with regular partners and men with casual partners, but their effects were different in the two groups.

The finding that access to condoms, as measured in this study, was not relevant to patterns of condom use is interesting; in bivariate and multivariate analysis among men with regular partners and men with casual partners, having cheap and convenient access to condoms had very little effect on condom use. This result is somewhat contrary to expectation, as lack of access to a condom was the second most commonly cited reason for non-use of condoms at last sex with a casual partner in the same study used for this analysis [12]. It is possible that relevance of condom access may not have been captured by asking about community locations for cheap and convenient condoms, i.e. that asking about condom access in the community does not correlate with having a condom available prior to sexual activity. This analysis also found that a relatively low proportion of men reported having access to a convenient location for cheap condoms (34%), compared to limited previous research in Africa that found that 82.5% and 63.5% of young men could locate condoms within a ten-minute walk [26], [27].

However, the importance of education in explaining condom use patterns is underscored by the fact that it was the variable with the largest magnitude of impact in the final multivariate models among men with regular partners and men with casual partners. The significance of education in explaining condom use patterns has been established by previous research [28], [29]. Similarly, among men with regular partners, being legally married was associated with much lower condom usage, a finding that is consistent with previous research [4], [30], where to use condom use with a regular partner is viewed as an insult to the partner [8] or representing infidelity [30].

In this analysis, condom-specific knowledge did not fully align with years of formal education; after adjusting for schooling, knowledge of condom effectiveness was found to be significantly associated with increased odds of reporting higher levels of condom use among both groups of men. The effect of knowing the condom’s role in HIV prevention was stronger among men with casual partners than among men with regular partners, which may be related to higher perceived HIV risk among men with casual partners. On the other hand, the higher level of condom use among men with regular partners had to be induced by a pro-condom strategy. To promote their condom use, one may have to change their condom attitudes first. This is not found in the case of men with casual partners.

Employment in lower-level jobs such as labor, sales and services, compared to professional jobs, was associated with decreased odds of reporting higher levels of condom use among men with regular partners. Particularly, laborers were found to be significantly associated with the lowest level of condom use. These results are similar to previous research establishing lower levels of condom use among laborers, farmers, and factory workers [28]. However, among men with casual partners, occupations in sales, service, or labor were associated with increased use of condoms, compared to the professional/technical occupations. This difference is intriguing; further research is warranted, and should also explore additional characteristics of the men’s partners in addition to selected characteristics of the men themselves. The study of the selectivity of men who were engaged in casual relationships would shed light to this discrepancy.

Among men with casual partners, shorter relationships were associated with more condom use, consistent with previous research and supporting the sawtooth hypothesis [5]. These men would be more aware of the risk of disease and concerned with pregnancy prevention with their “new” casual partners. In contrast, among men with regular partners, shorter relationships were associated with less condom use. On the one hand, these men and their partners may be selective of the most faithful, honeymoon period couples. On the other hand, they may have fertility intentions and want to start a family. In contrast to casual partners, and opposite to the sawtooth hypothesis, regular partners were perhaps more committed and probably had to employ trust strategy, even at the very beginning of their dedicated relationship. However, this finding deserves additional research.

Men with casual partners who had never paid for sex tended to use condoms more frequently than men who had paid for sex in the past. These men who never paid for sex may be more selective of those who were more conscious about safe sex and avoiding sexual risks. Among men with regular partners, a history of paying for sex within the past year did not reach significance in the proportional odds model, but was significant in the logistic model (analysis not shown here). This suggests that men with regular partners who recently paid for sex are more likely to use condoms in sexual relations with their regular partner. Further studies are needed to test whether it is possible that these men may still visit sex workers and/or may be aware of their possibility of infection.

Findings from this study help to formulate a framework for future studies of the dynamics of condom use among different partners. First, although the characteristics and motivation of engaging in casual sex is not a public health policy issue, understanding the selectivity of those who have extramarital and/or casual relation may provide important insight on the subsequent condom use behavior. Future studies should address all possible demographic and socioeconomic status, as control variables, when analyzing the dynamics of condom use patterns. At least age, urban/rural residence, and occupation should be investigated.

Second, “risk perception” factors (factors related to perception of risk of HIV/AIDS and STD) may be more relevant to condom use behavior than condom motivation factors. Risk perception is associated with formal education in general, but if data are available, the life skill knowledge and other informal training, in particular, should be included in the investigation as well. Most importantly, risk perception is also related to the perceived nature and type of relationship and partner characteristics. Under the theoretical framework of trust and fidelity, these factors include the degree of attachment in marriage and partnership, and the newness of relations. For regular partners, the higher level of attachment in marriage is associated with trust and fidelity and consequently less condom use. As for the newness of relation, again, according to the fidelity assumption, condom use is rarely seen during the honey moon period. The dynamism of condom behavior is that, for the casual partners, according to the sawtooth hypothesis, condom use is seen to be high during the first-meeting period of casual relation and will decline with duration and strength of relationship. How to keep risk perception of casual relation long-standing is the challenge of the intervention design. Lastly, perceived risk is also related to previous or current sexual experience and the primary person to protect from infection, self or partner. For regular partners, current experience of visiting sex worker (perceived probability of self infection) or perhaps having multiple partners, is associated with more condom use probably to protect their married or regular partners. In contrast, sex with casual partners was found to be more protected among men who did not have experience with sex workers. The protection is probably meant for these men themselves rather than for the protection of their partners.

Third, condom motivations or factors related to motivation to take preventive action by using condom should also be highlighted in the condom behavior framework. In this study knowledge of condom effectiveness in HIV prevention and attitude of condom use as a strategy to reduce HIV risk are found to be associated with higher levels of condom use. However, further studies on access to convenient and cheap condom sources are still needed. This is especially important since the public health intervention with appropriate and effective heath information messages, even in population where the majority of people are aware of condom effectiveness in preventing HIV, are still to be carefully designed.

The strengths of this analysis include the substantial sample size, drawn from a national probability sample of adults in Thailand, a country with substantial variation in condom usage due in part to a unique history of condom promotion messages. However, there were relatively few men who reported having a casual partner in the past year; this limitation hindered our ability to determine the true association between condom use and many variables of interest. Notably, less of the variance in condom use among men with casual partners was explained by the factors considered in this analysis. However, important results were drawn from the analysis of men with regular partners, confirming previous findings relating to the impact of marriage and education.

Clearly, more research is needed on the use of condoms during encounters with casual partners in Thailand. Particularly since HIV transmission through commercial sex has plummeted following the government’s 100% Condom Use program, HIV transmission through non-commercial partners is of increasing importance. Additionally, future studies should explore additional dimensions of condom access that may be more relevant in explaining condom use patterns. Exploring the determinants of perceived access to condoms may also be fruitful in identifying populations at risk and effective interventions to increase access to condoms. Apart from the issue of access, one should also take into account the dynamics of men’s decision or strategy to use or not to use a condom with different types of partners, with different stages of relationship, and in the family and non-family context. Self-perception of own risk of infections related to their previous or recent relationship with sex workers or other casual partners also shaped their condom use strategy with their current partners. Continued effort towards determining the factors that are associated with condom use among Thai males with their different types of partners, and in a variety of partnership circumstances, is crucial for designing appropriate and wide-ranging interventions to increase condom use and decrease transmission of HIV and other STIs.

Lastly, the findings from this study suggest that policy and interventions to promote condom use to prevent HIV/AIDs and STDs in Thailand need to take into account both the demand and supply side. That is, not only the availability and accessibility of condom information and services, but, in contrast to campaign on condom use with sex workers, the dynamics and sensitivities of condom use with more intimate partners have to be addressed. It is especially important to distinguish regular partners who can be just living together or more attached to each other by registered marriage. Casual partners who are not paid partners but have intimate relationship need to be delicately attended.

Risk perceptions of HIV/AIDS and STDs and motivation to preventive action among these partners are not straight forward and interact with partner intimacy and fidelity issues. First, the national HIV/AIDS prevention campaign should start with the fact that everyone has the risk, and that there are no specific risk groups, regardless of age and sex and inside or outside of marriage. Second, condom promotion should be desensitized by including the broader perspectives of health. The focus should be on total health issues including reproductive health and healthy family planning method for spacing, healthy childbirth, prevention of STIs where symptom of disease may not show. Condom campaign should also incorporate prevention against BV and HPV, where sexual relation (or at least current sexual relation) may not be involved. Third, the program should, at the same time, tackle the political, religious and community barriers concerning the sexual stigma in general and on casual and multiple partners. Intervention should address the gender bias especially on woman virginity and the family values that might overly stigmatize extra marital relations. Lastly, in general, condom campaign should be expressed in the terms of sanitation and health, intimacy, human relationship, family and caring rather than related to sexual diseases.
